# Primary Immunodeficiencies Unravel the Role of IL-2/CD25/STAT5b in Human Natural Killer Cell Maturation

**DOI:** 10.3389/fimmu.2018.01429

**Published:** 2018-06-22

**Authors:** María Soledad Caldirola, María Guadalupe Rodríguez Broggi, María Isabel Gaillard, Liliana Bezrodnik, Norberto Walter Zwirner

**Affiliations:** ^1^Servicio de Inmunología, Hospital de Niños “Ricardo Gutiérrez”, Buenos Aires, Argentina; ^2^Centro de Inmunología Clínica “Dra. Bezrodnik”, Buenos Aires, Argentina; ^3^Instituto de Biología y Medicina Experimental (IBYME-CONICET), Laboratorio de Fisiopatología de la Inmunidad Innata, Buenos Aires, Argentina; ^4^Departamento de Química Biológica, Facultad de Ciencias Exactas y Naturales, Universidad de Buenos Aires, Buenos Aires, Argentina

**Keywords:** natural killer cells, IL-2, CD25, STAT5b, primary immunodeficiencies

## Abstract

Natural killer (NK) cells play a pivotal role during immunity against viruses and circumstantial evidence also indicates that they can protect the host against developing tumors. Peripheral blood NK cells comprise CD56^bright^CD16^lo/−^ cells that constitutively express CD25 (IL-2Rα) and CD56^dim^CD16^hi^ cells that express CD25 upon activation. Using NK cells from two patients, one with a primary immunodeficiency characterized by a homozygous mutation in CD25 (born in year 2007 and studied since she was 3 years old) and one with a homozygous mutation in STAT5b (born in year 1992 and studied since she was 10 years old), we observed that the absence of IL-2 signaling through CD25 promotes the accumulation of CD56^bright^CD16^high^ NK cells, and that CD56^bright^CD16^lo^, CD56^bright^CD16^high^, and CD56^dim^CD16^high^ NK cells of this patient exhibited higher content of perforin and granzyme B, and proliferation capacity, compared to healthy donors. Also, CD56^bright^ and CD56^dim^ NK cells of this patient exhibited a reduced IFN-γ production in response to cytokine stimulation and increased degranulation against K562 cells. Also, the CD25-deficient patient presented a lower frequency of terminally differentiated NK cells in the CD56^dim^CD16^hi^ NK subpopulation compared to the HD (assessed by CD57 and CD94 expression). Remarkably, CD56^dim^CD16^high^ NK cells from both patients exhibited notoriously higher expression of CD62L compared to HD, suggesting that in the absence of IL-2 signaling through CD25 and STAT5b, NK cells fail to properly downregulate CD62L during their transition from CD56^bright^CD16^lo/−^ to CD56^dim^CD16^hi^ cells. Thus, we provide the first demonstration about the *in vivo* requirement of the integrity of the IL-2/CD25/STAT5b axis for proper human NK cell maturation.

## Introduction

Natural killer (NK) cells are essential players in immunity against viral infections (1–3). In addition, a prospective cohort study demonstrated that high cytotoxic activity of NK cells is associated with a decreased risk of cancer (4). They also display a high degree of diversity that determines their tissue tropism and responses to external insults (5, 6). Two major populations of NK cells can be detected in peripheral blood, based on the expression of CD56 and CD16 (FcRγIIIa): about a 90% are CD56^dim^CD16^hi^, while the rest exhibit a CD56^bright^CD16^lo/−^ phenotype. CD56^bright^CD16^lo/−^ cells are mostly cytokine producing-cells (in particular, IFN-γ), while CD56^dim^CD16^hi^ cells are mainly cytotoxic due to their high content of perforin and granzymes, but also produce cytokines (6, 7). CD56^bright^CD16^lo/−^ cells appear to be precursors of CD56^dim^CD16^hi^ NK cells (8–10). A phenotypic CD56^bright^CD16^hi^ intermediate stage in the progression of CD56^bright^ to CD56^dim^ NK cells that displays cytotoxic activity (ADCC and direct cytotoxicity) has been identified in peripheral blood of healthy donors (HDs) and patients after hematopoietic stem cell transplantation (HSCT) (9–13). Moreover, CD56^bright^CD16^lo/−^ (abundant in lymph nodes) can differentiate into CD56^dim^CD16^hi^-like cells (abundant in efferent lymphatics) in the presence of IL-2 and autologous T cells, suggesting that these subpopulations are developmentally connected (10, 12, 13). However, the physiological stimuli that promote such differentiation remain undefined. Also, NK cells can be divided into developmental stages based on the expression of CD27 and CD11b (14–18).

CD56^bright^CD16^lo/−^ NK cells constitutively express the high affinity IL-2 receptor (IL-2R), a trimeric receptor composed of CD25 (α chain), CD122 (β chain), and CD132 (common γ chain) (10, 19–21), while CD56^dim^CD16^hi^ NK cells constitutively express the low affinity IL-2R, a dimeric receptor composed of CD122 and CD132 (21). CD25 is also induced in CD56^dim^ cells upon activation by cytokines, making them more responsive to IL-2 (22, 23). In turn, IL-2 signals through activation of Jak1/Jak3 kinases that activate STAT5a/STAT5b-dependent signaling pathways (24). However, IL-2 can also promote some effects in a STAT5b-independent manner through the activation of STAT1α and STAT3 in primary T cells (25, 26). Moreover, STAT5a and STAT5b mediate partially overlapping functions but also different functions in human T cells (27). Remarkably, the role of IL-2 in human NK cell maturation remains unknown, mostly due to the lack of suitable genetic deficient individuals that may shed light on this issue.

Human inborn genetic primary immunodeficiencies (PID) that affect the generation, homeostasis, and/or function of NK cells constitute straightforward models to understand human NK cell immunobiology (1, 28). Among over 300 known genetic deficiencies, nearly 50 impact on NK cells (29–34). Human CD25 deficiency, caused by mutation in the *IL2RA* gene, is a combined immunodeficiency characterized by invasive viral and bacterial sinopulmonary infections, lymphoproliferation, and severe multi-organ autoimmune disorders (35). Only four CD25 deficient patients have been reported, and very little is known about the consequences of CD25 deficiency on the NK cell compartment (30, 36–38). Moreover, STAT5b deficiency also is a rare PID with only 10 cases described, some of which are associated with high susceptibility to varicella and herpes virus infections (39).

Considering that these deficiencies may affect NK cells and determine the clinical picture of the patients, we performed a characterization of NK cells in one patient with a homozygous CD25 deficiency and in one patient with a homozygous STAT5b deficiency, both of which have been previously described by our group (38, 40, 41). We unraveled a critical role of the IL-2/CD25/STAT5b axis in NK cell maturation and partially explain the clinical symptoms of the patients, re-emphasizing the critical role of NK cells in immunity.

## Materials and Methods

### Samples

Two patients were included in this study. Patient 1, born in year 2007 and studied since she was 3 years old, carries a homozygous missense mutation that introduces an amino acid substitution in position 41 of the extracellular domain of CD25 (Y41S) that abrogates its expression without affecting expression of CD122 and CD132. This patient presented severe atopic dermatitis, chronic diarrhea, and several respiratory infections, associated with chronic and severe inflammatory lung disease (follicular bronchiolitis with lymphocyte hyperplasia), eczema, and infections (in particular, a severe varicella) (38). Patient 2, born in year 1992 and studied since she was 10 years old, carries a homozygous missense mutation that introduces an amino acid substitution (F646S) in the βD′ strand of the SH2 domain of STAT5b. This patient presented upper and lower respiratory tract recurrent infections, severe cutaneous eczema, episodic infections in the first years of life, autoimmune thyroiditis, and pronounced growth failure (41). Whole blood from the patients and from HDs was collected with EDTA or heparin. Blood collection was performed when the patients were clinically stable (with no signs of infections or other major health conditions directly perceptible by the physician). In some cases, peripheral blood mononuclear cells (PBMCs) were isolated by Histopaque^®^ 1077 (Sigma) centrifugation and cultured in RPMI 1640 (Sigma) supplemented with 10% inactivated fetal bovine serum (Invitrogen), glutamine, gentamicin, and penicillin. Samples from age-matched HD attending the Immunology Unit from the “Ricardo Gutierrez” Children’s Hospital (Buenos Aires, Argentina) were also used. Studies have been approved by the institutional review committee and informed and written consent of the parents of the participating subjects were obtained.

### Antibodies and Reagents

The following monoclonal antibodies (mAb) against human molecules were used: PE-anti-NKp46 (9E2); PE-anti-NKG2D (1D11), PerCP/Cy5.5-anti-CD16 (3G8), FITC-anti-CCR7 (G043H7), Alexa488-anti-perforin (δG9), PE-anti-Granzyme B (GB11), PE-anti-IFN-γ (4S.B3), FITC-anti-T-bet (4B10), PE-anti-CD11b (ICRF44), and PE-Cy7-anti-CD3 (UCHT-1), FITC-anti-CD27 (M-T271), PE-Cy7-anti-CD94 (DX22) and PE-anti-IL-18Rα (H44) from Biolegend; PE-anti-CD25 (2A3), PE-anti-CD62L (SK11), PE-Cy5 anti-CD107a (H4A3), FITC-anti-CD57 (NK-1), APC-anti-IL-12Rβ1 (2.4E6), PE-anti-12Rβ2 (2B6/12beta2) and PE-Cy5 mouse IgG1κ (MOPC-21, isotype-matched control mAb; IC) from BD; APC-anti-CD56 (N901) from Beckman Coulter; and PE-anti-IL-18Rβ (132029) from R&D Systems. Human rIL-12 (PeproTech), rIL-15 (PeproTech), rIL-18 (MBL), and rIL-2 (Proleukin^®^, Prometheus) were also used.

### Flow Cytometry

Immunostaining was performed using whole blood or PBMC. For whole blood, 100 µl of blood collected with EDTA were stained during 15 min at room temperature with the mAb. Thereafter, red blood cells were lysed using FACSLysing solution (BD) for 7 min, washed with PBS, and acquired. For PBMC, 5 × 10^5^ cells were stained with the mAb for 15 min, washed with PBS, and acquired. Expression of IFN-γ and T-bet was analyzed by intracellular flow cytometry (FC) using Cytofix/Cytoperm (BD) following manufacturer’s protocol. For IFN-γ, cells were cultured in the presence of Golgi-Stop^®^ during the last 4 h. For perforin and Granzyme B expression, Dako Intrastain kit was used. Cells were acquired in a FACSCanto II flow cytometer (BD) and analyzed using FlowJo (Treestar, Inc.). Negative populations were established with the “fluorescence minus one” tube (FMO). Results were expressed as MFI. Prism 5.0 (GraphPad Software) was used to plot the results.

### NK Cell Proliferation

Peripheral blood mononuclear cells were stained with Carboxyfluorescein succinimidyl ester (CFSE, CellTrace™ CFSE Proliferation Kit- Invitrogen, Molecular Probes^®^) and cultured in the absence or in the presence of rIL-15 (2 ng/ml) and rIL-2 (30 U) during 5 days at 37°C with 5% of CO_2_. Proliferation was assessed as the frequency of dividing cells (% of CFSE^low^).

### IFN-γ Production

Peripheral blood mononuclear cells were cultured overnight in the absence or in the presence of rIL-12 (10 ng/ml), rIL-18 (10 ng/ml), and rIL-15 (2 ng/ml). During the last 4 h, Golgi-Stop^®^ (BD) was added to the cultures and IFN-γ production was assessed by FC as described (23). NK cells were gated as CD3^−^CD56^+^ cells.

### NK Cell Degranulation Assay

Peripheral blood mononuclear cells were cultured for 3 h without or with K562 target cells (effector:target ratio: 1:3) at 37°C with 5% of CO_2_ in the presence of the anti-human CD107a mAb or an IC mAb. Thereafter, NK cell degranulation was analyzed by FC. NK cells were gated as CD3^−^CD56^+^ cells, and degranulation was expressed as percentage of CD107a^+^ NK cells. Background degranulation of unstimulated cells (without target cells) was always below 5%.

## Results

### CD25 Deficiency Promotes the Accumulation of Dysfunctional NK Cells That Contain an Increased Frequency of CD56^bright^CD16^hi^ Cells

**Figure 1:**
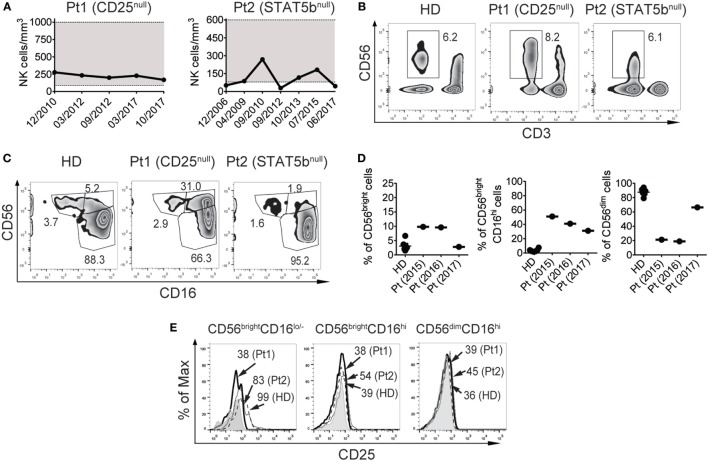
CD25 deficiency leads to an increased frequency of CD56^bright^CD16^hi^ natural killer (NK) cells and a concomitant decreased frequency of CD56^dim^CD16^hi^ NK cells in peripheral blood. **(A)** Absolute NK cell numbers in blood from the CD25-deficient patient (left graph) and the STAT5b-deficient patient (right graph) along time (indicated as month/year). Horizontal dotted lines delineate a gray zone that corresponds to the reference values for age-matched healthy donors (HDs). **(B)** Percentage of NK cells (CD3^−^CD56^+^ cells) in peripheral blood from one HD, the CD25-deficient patient (Pt1), and the STAT5b-deficient patient (Pt2). **(C)** Percentage of NK cell subpopulations according to the expression of CD56 and CD16, in peripheral blood from one HD, the CD25-deficient patient (Pt1) and the STAT5b-deficient patient (Pt2). **(D)** Percentage of CD56^bright^CD16^lo/−^, CD56^bright^CD16^hi^, and CD56^dim^CD16^hi^ cells in blood from different HD, and in different blood samples from the CD25-deficient patient collected along three years (2015, 2016, and 2017). **(E)** Analysis of the expression of CD25 in CD56^bright^CD16^lo/−^, CD56^bright^CD16^hi^, and CD56^dim^CD16^hi^ cells from one HD (dashed histograms), in the CD25-deficient patient (continuous black histograms) and in the STAT5b-deficient patient (dotted histograms). Gray histograms: FMO. Numbers in the zebra plots from **(B,C)** correspond to the percentages of each gated cell population; numbers in the histograms from **(E)** correspond to MFI. Results shown in **(B,C,E)** representative of three independent blood samples collected over the years. Pt1 began to be studied when she was 3 years old (year 2010). Pt2 began to be studied when she was 10 years old (year 2010). Data shown in **(B,C,E)** correspond to blood samples obtained in year 2017 (when Pt1 was 10 years old and Pt2 was 25 years old).

The CD25-deficient systematically exhibited NK cell counts within the range of the healthy population (221 ± 41 NK cells/mm^3^) since she was 3 years old (when she began to be studied in year 2010), while the STAT5b-deficient patient exhibited low NK cell counts sporadically (average: 111 ± 87 NK cells/mm^3^) since she was 10 years old (when she began to be studied in year 2002) (Figure 1A). In terms of frequencies, these values were 4.8 ± 2.3% of NK cells in the CD25-deficient patient and 13.4 ± 7.5% in the STAT5b-deficient patient (representative zebra plots of CD3^−^CD56^+^ cells are shown in Figure 1B). Moreover, the CD25-deficient patient, but not the STAT5b-deficient patient, exhibited a large increased frequency of CD3^−^CD56^bright^CD16^hi^ NK cells and an accompanying decrease in the frequency of CD3^−^CD56^dim^CD16^hi^ NK cells (Figure 1C). For the CD25-deficient patient (who never exhibited NK cell numbers below the reference values), these frequencies were relatively stable along time (Figure 1D). Also, only CD3^−^CD56^bright^CD16^lo/−^ NK cells from the HD and from the STAT5b-deficient patient but not from the CD25-deficient patient, expressed CD25 (Figure 1E). Therefore, the absence of CD25 (but not the absence of STAT5b) promotes the accumulation of less mature NK cells with a CD56^bright^CD16^hi^ phenotype.

**Figure 2:**
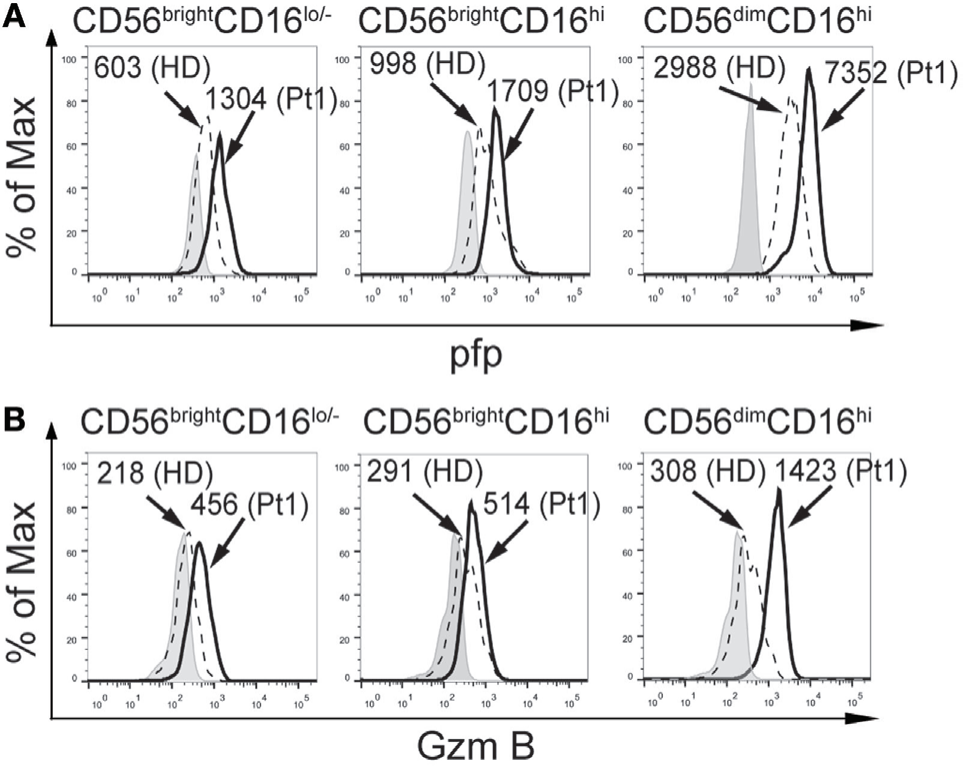
CD25 deficiency leads to higher amounts of perforin and granzyme B expression in natural killer cell subpopulations. Analysis of the expression of perforin (pfp), **(A)** and granzyme B (GzmB) **(B)**, in CD56^bright^CD16^lo/−^, CD56^bright^CD16^hi^, and CD56^dim^CD16^hi^ cells from one HD (dashed histograms) and in the CD25-deficient patient (continuous black histograms). Gray histograms: FMO. Numbers in the histograms correspond to MFI. Results are representative of three independent blood samples collected over the years.

Next, we analyzed the expression of some molecules critically involved in NK cell effector functions. Although we did not observe differences NKp46 and NKG2D expression in the CD56^bright^CD16^lo/−^, CD56^bright^CD16^hi^, and CD56^dim^CD16^hi^ subpopulations of NK cells between HD and the CD25-deficient patient (*not shown*), the three NK cell subpopulations from the CD25-deficient patient contained higher amounts of perforin (Figure 2A) and granzyme B (Figure 2B) compared to HD. These results suggest that the IL-2/CD25 signaling is dispensable for the normal synthesis and expression of these major lytic mediators and that this signaling pathway might function as negative regulator of their expression.

**Figure 3:**
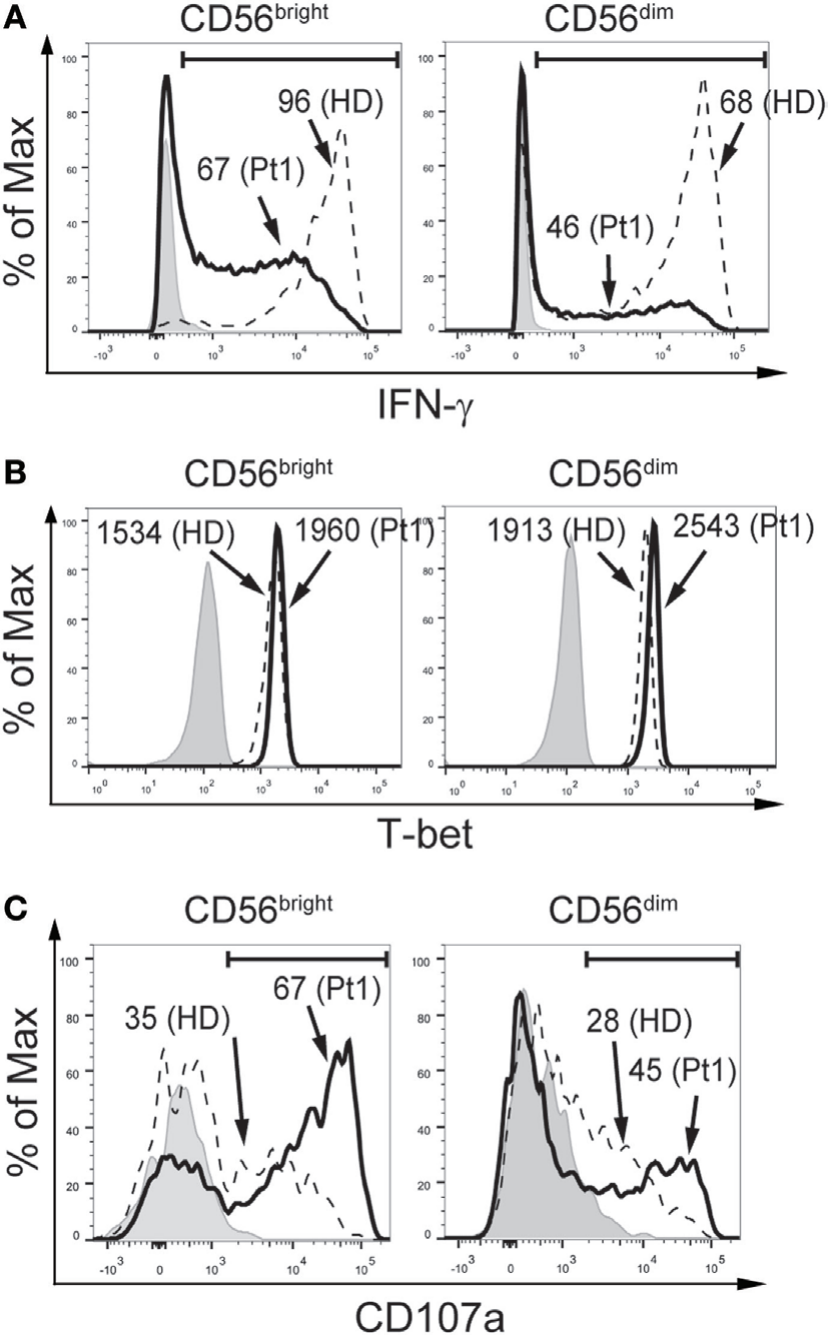
CD25 deficiency leads to impaired IFN-γ production but does not negatively affect degranulation by CD56^bright^ and CD56^dim^ natural killer (NK) cells. **(A)** Percentage of IFN-γ-producing CD56^bright^ (left histograms) and CD56^dim^ NK cells (right histograms) from one HD (dashed histograms) and from the CD25-deficient patient (continuous black histograms) in response to stimulation with IL-12, IL-15, and IL-18. **(B)** Percentage of T-bet-expressing CD56^bright^ (left histograms) and CD56^dim^ NK cells (right histograms) from one HD (dashed histograms) and from the CD25-deficient patient (continuous black histograms). **(C)** Degranulation of CD56^bright^ (left histograms) and CD56^dim^ NK cells (right histograms) from one HD (dashed histograms) and from the CD25-deficient patient (continuous black histograms) in response to stimulation with K562 cells. In all panels, gray histograms correspond to unstimulated cells. Numbers in the histograms from **(A,C)** correspond to the percentage of positive cells; numbers in the histograms from **(B)** correspond to MFI. Results are representative of three independent blood samples collected over the years.

Additionally, we assessed the functional response of NK cells from the CD25-deficient patient (Figure 3). CD56^bright^ and CD56^dim^ NK cells from the CD25-deficient patient produced less IFN-γ than CD56^bright^ and CD56^dim^ NK cells from the HD (Figure 3A). However, CD56^bright^ and CD56^dim^ NK cells from the CD25-deficient patient expressed slightly higher amounts of T-bet than CD56^bright^ and CD56^dim^ NK cells from the HD (Figure 3B). Moreover, CD56^bright^ and CD56^dim^ NK cells from the CD25-deficient patient displayed an increased degranulation when compared to the HD (Figure 3C). Of note, the impaired IFN-γ production of CD56^bright^ and CD56^dim^ NK cells from the CD25-deficient patient was not due to an inability to sense the cytokines used for the stimulation as we did not observe differences in the expression of IL-12Rβ1 and IL-12β2 between HD and the CD25-deficient patient in each of the three NK cell subpopulations (*not shown*), the expression of CD122 and CD132 is not affected in the patient (38), and we detected a slightly higher expression of IL-18Rα and IL-18Rβ in each of the three NK cell subpopulations from the CD25-deficient patient compared to the three NK cell subpopulations from HD (*not shown*).

### CD56^bright^CD16^hi^ NK Cells Accumulated in CD25- or STAT5b-Deficient Patients Display a Not Fully Mature CD62L^hi^ Phenotype

**Figure 4:**
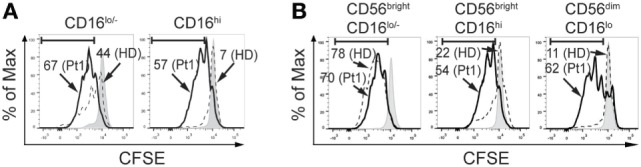
CD25 deficiency leads to increased proliferative response of natural killer (NK) cells. **(A)** Percentage of CFSE^low^ NK cells from one HD (dashed histograms) and in the CD25-deficient patient (continuous black histograms) in response to stimulation with IL-2 plus IL-15 in CD16^lo/−^ NK cells (left histograms) and in CD16^hi^ NK cells (right histograms). **(B)** Percentage of CFSE^low^ NK cells from one HD (dashed histograms) and in the CD25-deficient patient (continuous black histograms) in response to stimulation with IL-2 plus IL-15 in CD56^bright^CD16^lo/−^ NK cells (left histograms), in CD56^bright^CD16^hi^ NK cells (middle histograms) and in CD56^dim^CD16^hi^ NK cells (right histograms). Numbers in the histograms correspond to the percentage of CFSE^low^ NK cells. Gray histograms: CFSE-labeled NK cells cultured in the absence of IL-2 and IL-15. Results are representative of three independent blood samples collected over the years.

The increased frequency of CD56^bright^CD16^hi^ NK cells observed in the patient with CD25 deficiency suggests an arrest in NK cell maturation. As maturation is usually associated with a decreased proliferative capacity, we assessed proliferation of CD16^lo/−^ (less mature) and CD16^hi^ (more mature) NK cells in response to IL-2 and IL-15, as it is difficult to assess proliferation in CD56^bright^CD16^lo/−^, CD56^bright^CD16^hi^, and CD56^dim^CD16^hi^ NK cell subsets because CD56 is upregulated during NK cell activation and CD56^dim^ cells become CD56^hi^ cells during the stimulation period. We observed that CD16^lo/−^ NK cells from HD exhibited an intense proliferation (44% of CFSE^low^ cells) but CD16^lo/−^ NK cells from the CD25-deficient patient exhibited an even higher proliferation (67% of CFSE^low^ cells for the patient; Figure 4A, left graph). Moreover, CD16^hi^ NK cells from HD exhibited very low proliferation (7% of CFSE^low^ cells), while CD16^hi^ NK cells from the CD25-deficient patient exhibited an intense proliferation (57% of CFSE^low^ cells; Figure 4A, right graph). Therefore, both CD16^lo/−^ (less mature) and CD16^hi^ (more mature) NK cells cell from the CD25-deficient patient maintain a high proliferative response. Moreover, as the higher proliferation detected in the CD16^hi^ population can be due to the increased frequency of CD56brightCD16hi NK cells present in the CD25 deficient patient, we re-analyzed the proliferation data and gated the cells based on CD56 and CD16 expression (besides the limitation mentioned above regarding the upregulation of CD56 that occurs during NK cell activation). We observed that CD56^bright^CD16^lo^ NK cells from the patient proliferated similarly as CD56^bright^CD16^lo^ NK cells from the HD (Figure 4B, left graph). However, both, CD56^bright^CD16^hi^ and CD56^dim^CD16^hi^ NK cells from the patient exhibited a markedly increased proliferation compared to the equivalent NK cell subpopulations from the HD (Figure 4B, middle and right graph). These results indicate that these more mature NK cells from the CD25-deficient patient do not loose proliferative capacity when they differentiate into more mature CD56^bright^CD16^hi^ and CD56^dim^CD16^hi^ NK cells, which confirms the occurrence of a maturation defect in the NK cell compartment of the CD25-deficient patient.

**Figure 5:**
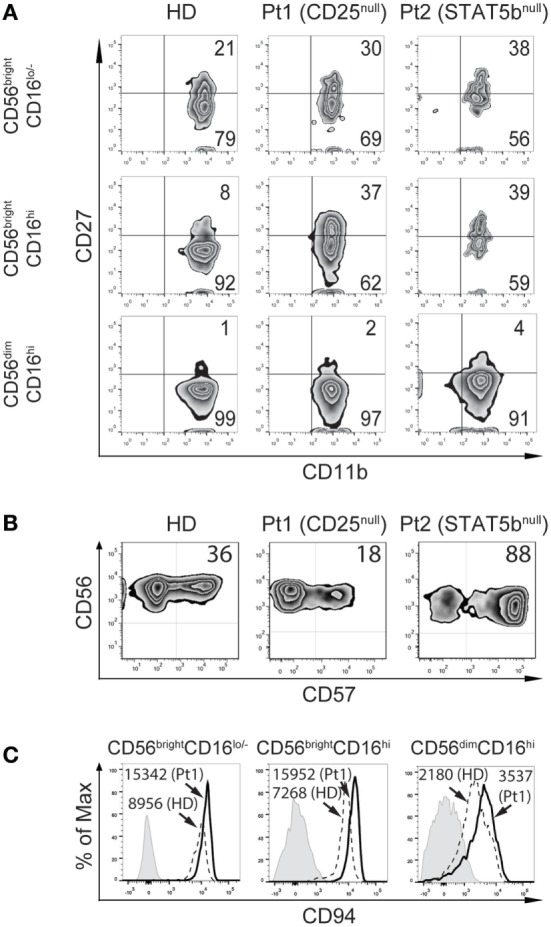
CD25 and STAT5b deficiencies leads to accumulation of CD27^+^CD11b^+^ natural killer (NK) cells with different consequences on the frequency of CD57^+^CD56^dim^CD16^hi^ NK cells. **(A)** Analysis of maturation stages according to the expression of CD27 and CD11b in CD56^bright^CD16^lo/−^, CD56^bright^CD16^hi^, and CD56^dim^CD16^hi^ NK cells from peripheral blood from one healthy donor (HD), the CD25-deficient patient (Pt1), and the STAT5b-deficient patient (Pt2). **(B)** Analysis of the expression of CD57 in CD56^dim^CD16^hi^ NK cells from peripheral blood from one HD, the CD25-deficient patient (Pt1) and the STAT5b-deficient patient (Pt2). Numbers in the zebra plots correspond to the percentages of each cell population. **(C)** Analysis of the expression of CD94 in CD56^bright^CD16^lo^, CD56^bright^CD16^hi^, and CD56^dim^CD16^hi^ NK cells from peripheral blood from one HD (dashed histograms) and the CD25-deficient patient (continuous black histograms). Gray histograms: FMO. Numbers in the histograms correspond to MFI.

Furthermore, we performed an analysis of the expression of CD27 and CD11b on CD56^bright^CD16^lo/−^, CD56^bright^CD16^hi^, and CD56^dim^CD16^hi^ cells (Figure 5A). NK cells from the CD25-deficient and from the STAT5b-deficient patients contained an increased frequency of CD27^+^CD11b^+^ cells with a concomitant decreased frequency of more differentiated CD27^−^CD11b^+^ NK cells, both in the CD56^bright^CD16^lo/−^ and in the CD56^bright^CD16^hi^ NK cell subsets. Conversely, the distribution of CD27^+^CD11b^+^ and CD27^−^CD11b^+^ NK cells within the more mature CD56^dim^CD16^hi^ cells were similar in the deficient patients compared to the HD. Nonetheless, these distributions of cells based on the expression of CD27 and CD11b is in the context of different absolute NK cell numbers (228 NK cells/mm^3^ for the CD25-deficient patient and 45 NK cells/mm^3^ for the STAT5b-deficient patient), suggesting that both mutations lead to a different abnormal maturation program in NK cells.

Moreover, we detected a lower frequency of CD57^+^ cells (cells with features of terminally differentiated NK cells) in the CD56^dim^CD16^hi^ NK cells in the CD25-deficient patient compared to the HD, and an increased frequency of CD57^+^ cells in the STAT5b-deficient patient (Figure 5B). Moreover, the CD25-deficient patient displayed higher expression of CD94 in each NK cell subpopulation, compared to HD (Figure 5C). These results confirm the occurrence of a defective maturation in the absence of proper IL-2 signaling through its high affinity receptor.

**Figure 6:**
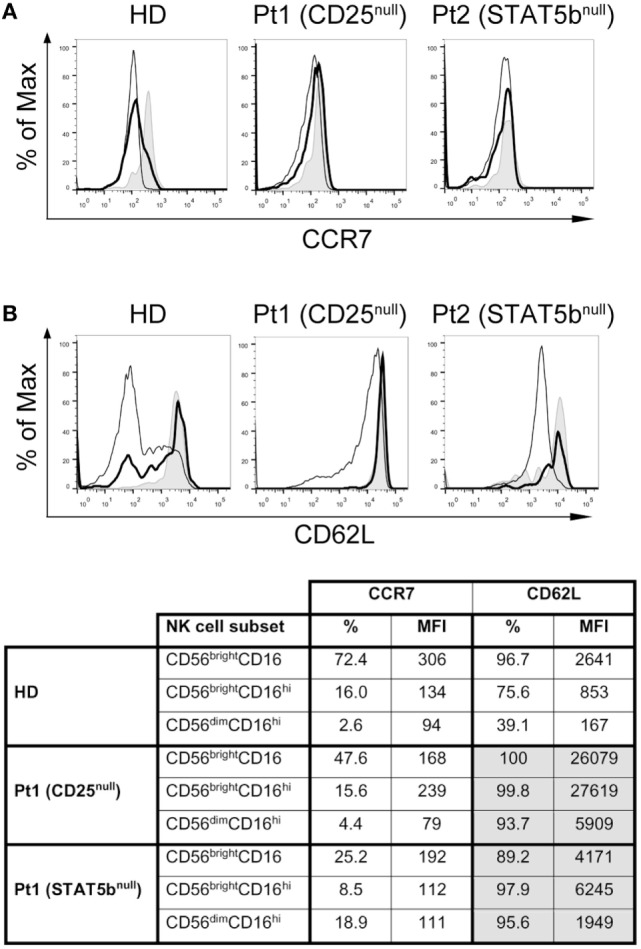
CD25 and STAT5b deficiencies do not affect CCR7 expression, but negatively affect downregulation of CD62L on CD56^bright^CD16^hi^ and CD56^dim^CD16^hi^ natural killer cell subpopulations. Analysis of the expression of CCR7 **(A)** and CD62L **(B)** on CD56^bright^CD16^lo/−^ cells (gray histograms), CD56^bright^CD16^hi^ cells (continuous thick line), and CD56^dim^CD16^hi^ cells (continuous thin lines) from one HD, in the CD25-deficient patient (Pt1) and in the STAT5b-deficient patient (Pt2). The appended table summarizes the percentage and MFI for CCR7 and CD62L on each cell population from each sample.

As the transition of CD56^bright^ to CD56^dim^ cells is associated with the downregulation of CCR7 and CD62L, and a loss of lymph node homing potential, we explored their expression in the NK cell subpopulations from HD and the two immunodeficient patients (Figure 6). We observed a slightly lower expression of CCR7 (in terms of frequency of positive cells and MFI) in CD56^bright^CD16^lo/−^ cells from the CD25-deficient and the STAT5b-deficient patients compared to the HD (Figure 6A and inserted table), suggesting a partial requirement of the IL-2/CD25/STAT5b axis for the expression of CCR7 on this NK cell subset. No major differences in the expression of CCR7 were observed in CD56^bright^CD16^hi^ and CD56^dim^CD16^hi^ cells between HD and the patients. Conversely, CD56^bright^CD16^lo/−^ cells from the CD25-deficient patient expressed much higher amounts of CD62L compared to HD or the STAT5b-deficient patient (Figure 6B and inserted table). Moreover, while NK cells from the HD progressively downregulated CD62L along maturation stages (from CD56^bright^CD16^lo/−^ to CD56^bright^CD16^hi^ to CD56^dim^CD16^hi^ cells), such downregulation was not observed in NK cells from the CD25-deficient and from the STAT5b-deficient patient, as even CD56^dim^CD16^hi^ NK cells from both displayed high amounts of CD62L. These results suggest that the absence of adequate IL-2 signaling impedes the downregulation of CD62L that is usually a feature observed during the transition of CD56^bright^CD16^lo/−^ to CD56^dim^CD16^hi^ NK cells.

## Discussion

Natural killer cells develop from a bone marrow common lymphoid progenitor and complete their differentiation in different niches including lymph nodes and peripheral blood, where they acquire functional competence to respond to pathogens and tumor cells (5). Human NK cell maturation differs in many aspects from mouse NK cell maturation, and several aspects of human NK cell development remain ill-defined. Among them, we can mention the role of the expression of the high affinity IL-2 receptor on CD56^bright^CD16^lo/−^ cell immunobiology. Taking advantage of the fact that human patients with PID constitute “*natural models*” of genetic deficiencies that provide an outstanding opportunity to elucidate still unknown features of the immune system (1, 28, 33, 34), here, we described the phenotypic and functional characteristics of the NK cells of a patient with a homozygous CD25 deficiency and compared them with NK cells from a patient with a STAT5b homozygous deficiency and HDs. CD25 deficiency did not affect NK cell frequency and absolute numbers but lead to an accumulation of CD56^bright^CD16^hi^ NK cells, with a concomitant decreased frequency of CD56^dim^CD16^hi^ NK cells in peripheral blood. These phenotypic alterations were not observed in the STAT5b-deficient patient, indicating that STAT5b signaling is dispensable but IL-2/CD25 signaling is necessary for the generation of fully mature CD56^dim^CD16^hi^ NK cells.

In addition, NK cells from the CD25-deficient patient displayed higher expression of perforin and granzyme B, suggesting that the IL-2/CD25 signaling pathway constitutes a negative regulator of the expression of these lytic mediators. Also, NK cells from the CD25-deficient patient displayed increased degranulation. Therefore, tonic signaling of IL-2 may fine-tune the content of perforin and granzyme B, and the degranulation capacity of NK cells. Conversely, NK cells from the CD25-deficient patient exhibited an impaired IFN-γ production with no alterations in the expression of T-bet, a critical transcription factor necessary for its production (42). However, the CD25-deficient and the STAT5b-deficient patients did not exhibit increased susceptibility of infection with mycobacteria (43). Therefore, our results unravel an important role of the IL-2/CD25 axis in the calibration of the effector function capacity of NK cells.

Natural killer cell deficiencies are associated with severe viral infections, caused predominantly by members of the *Herpesviridae* and *Papillomaviridae* families, but there is also evidence that they play an important role in immunity against a broader spectrum of viruses (1). In this study, we analyzed NK cells from a previously described CD25-deficient patient that carries a homozygous missense mutation in the *IL-2RA* gene that abrogates the expression of CD25 (38). This patient presented severe atopic dermatitis, chronic diarrhea, and several respiratory infections that began at 6 months of age that required frequent hospitalization. She also developed severe varicella infection that was treated with acyclovir. After being discharged, she developed alopecia, continued with bronchospasms, several lower and upper respiratory infections and exacerbations of her dermatitis. She also developed torpid pneumonia needing permanent oxygen therapy at the age of 4. A lung biopsy revealed that she suffered follicular bronchiolitis with lymphocyte hyperplasia. She was treated with corticosteroids, antibiotic prophylaxis, rapamycin, and intravenous gammaglobulin, and her condition improved, making oxygen therapy no longer necessary. Currently, she remains clinically stable and continues to receive this combined treatment waiting for bone marrow transplantation. In line with the viral infections suffered, we also observed abnormalities in her NK cells. The functional abnormalities of NK cells from this patient (compromised IFN-γ production without impaired degranulation and content of lytic mediators) could be partially responsible for her clinical picture, in particular, the varicella infection that she suffered. In addition, we also used NK cells from another previously described patient that carries a homozygous missense mutation in STAT5b (41). This patient presented several episodes of infection (otitis media, cellulitis, and pneumonia) during her first 3 months of life, developed severe generalized seborrheic dermatitis and autoimmune thyroiditis when she was 1 year old, psoriasis and alopecia at the age of 4, severe varicella with cutaneous infection caused by Streptococcus pyogenes, and celiac disease when she was 20 years old. Currently, she has persistent secondary psoriasis dermatitis and hypothyroidism, but no signs or symptoms of lung disease, and remains free of treatment (besides the hormone replacement treatment that she receives for her autoimmune thyroiditis; she never received prophylactic gammaglobulin treatement). In this patient, we also observed abnormalities in her NK cells.

The CD25-deficient patient, but not the STAT5b-deficient patient, exhibited an increased frequency of an infrequent CD56^bright^CD16^hi^ NK cell subset in peripheral blood. Such cells have been detected in efferent lymphatics and can be generated upon stimulation of CD56^bright^CD16^lo/−^ NK cells with IL-2 and autologous T cells (10, 12, 13). Therefore, the presence of these NK cells in peripheral blood of the CD25-deficient patient strongly point to the IL-2/CD25 pathway as a major physiological pathway that determines the transition from CD56^bright^CD16^lo/−^ to CD56^dim^CD16^hi^ NK cells. Moreover, CD16^lo/−^ and CD16^hi^ NK cells from the CD25-deficient patient exhibited an increased proliferation, which is a characteristic of less mature NK cells (13). In the case of the CD16^lo^ NK cells, we can speculate that this higher proliferative capacity might be the consequence of the deficient signaling of IL-2 during NK cell maturation. It is possible that when IL-2 signals through its high affinity receptor, NK cells become fine-tuned to optimally express molecules such as perforin, granzyme B, T-bet, and acquire an adequate maturation program. Moreover, although the higher proliferation of CD16^hi^ NK cells from the CD25-deficient patient could be the consequence of the increased frequency of CD56^bright^CD16^hi^ NK cells present in that population, both CD56^bright^CD16^hi^ and CD56^dim^CD16^hi^ NK cells from the CD25-deficient patient exhibited a markedly increased proliferation. These results indicate that these NK cells from the CD25-deficient patient do not loose proliferative capacity when they differentiate into more mature CD56^bright^CD16^hi^ and CD56^dim^CD16^hi^ NK cells, which probably underlies a defective maturation. Moreover, we also detected a lower frequency of CD57^+^ cells in CD56^dim^CD16^hi^ NK cells and a defective downregulation of CD94 in the different NK cell maturation stages in the CD25-deficient patient [cells with features of terminally differentiated NK cells (44–47)], further supporting the notion that CD25-deficiency leads to a defective maturation of NK cells in periphery.

Of note, as STAT5b is also a critical mediator of IL-15 signaling as it constitutes a downstream mediator of CD122 (26), the markedly increased frequency of CD57^+^CD56^dim^CD16^hi^ NK cells detected in the STAT5b-deficient patient may be the consequence of an abnormal signaling by IL-15 during NK cell maturation in the bone marrow, which would affect the generation of NK cells in peripheral blood (the STAT5b-deficient patient sporadically displayed reduced numbers of NK cells in blood, as mentioned earlier). Therefore, the few NK cells that this patient can produce display features of terminally differentiated cells. However, it is possible that they actually might be senescent NK cells as CD57 has been described as a marker of senescent T cells (48). Accordingly, both patients exhibited an increased frequency of CD27^+^CD11b^+^ NK cells with a concomitant decreased frequency of CD27^−^CD11b^+^ cells within the CD56^bright^CD16^lo/−^ and the CD56^bright^CD16^hi^ NK cell subsets, despite differences in the absolute NK cell numbers in both patients. Altogether, these results confirm the occurrence of a maturation defect in the absence of proper IL-2/CD25/STAT5b signaling, although mutations in CD25 or in STAT5b affect the maturation program of NK cells in a different manner.

Downregulation of CCR7 and CD62L is part of the normal NK cell transition from CD56^bright^CD16^lo/−^ to CD56^dim^CD16^hi^ cells (49–51). We observed that the IL-2/CD25/STAT5b is dispensable for the downregulation of CCR7 during the maturation of CD56^bright^CD16^lo/−^ to CD56^dim^CD16^hi^ cells. Moreover, this signaling route seems to act as negative regulator that controls the basal expression of CD62L in CD56^bright^CD16^lo/−^ cells as deficient NK cells subsets expressed higher amounts of CD62L than NK cells from HDs. Therefore, tonic signaling of IL-2 seems to be necessary to fine-tune the expression of CD62L on NK cells. Also, the IL-2/CD25/STAT5b axis appears to be critical for the downregulation of CD62L during the transition from CD56^bright^CD16^lo/−^ cells to CD56^bright^CD16^hi^ cells and, later, to fully mature CD56^dim^CD16^hi^ cells. Thus, the IL-2/CD25/STAT5b pathway indirectly regulates the adhesion potential of CD56^bright^CD16^lo/−^ NK cells to the CD62L ligands expressed on HEV, and the subsequent homing into lymph nodes.

Physiologically, the IL-2 that would regulate the transition of CD56^bright^CD16^lo/−^ to CD56^dim^CD16^hi^ cells is derived from activated T cells and dendritic cells in the paracortical areas of the lymph nodes (where CD56^bright^CD16^lo/−^ are located). Therefore, although IL-2 has been shown to promote the differentiation of CD56^bright^CD16^lo/−^ NK cells into CD56^dim^CD16^hi^ NK cells (10) *in vitro*, our studies with CD25-deficient and STAT5b-deficient NK cells provide the first formal proof of the involvement of IL-2 in NK cell maturation in physiological conditions. Our results also indicate that upregulation of the CD16 molecule does not require this IL-2/CD25/STAT5b signaling, as increased frequencies of CD16^hi^ NK cells were detected in the CD25-deficient and in the STAT5b-deficient patients. Recently, expression of human CD16 has been associated with epigenetic regulatory mechanisms negatively controlled miR-218 in NK cells (52).

**Figure 7:**
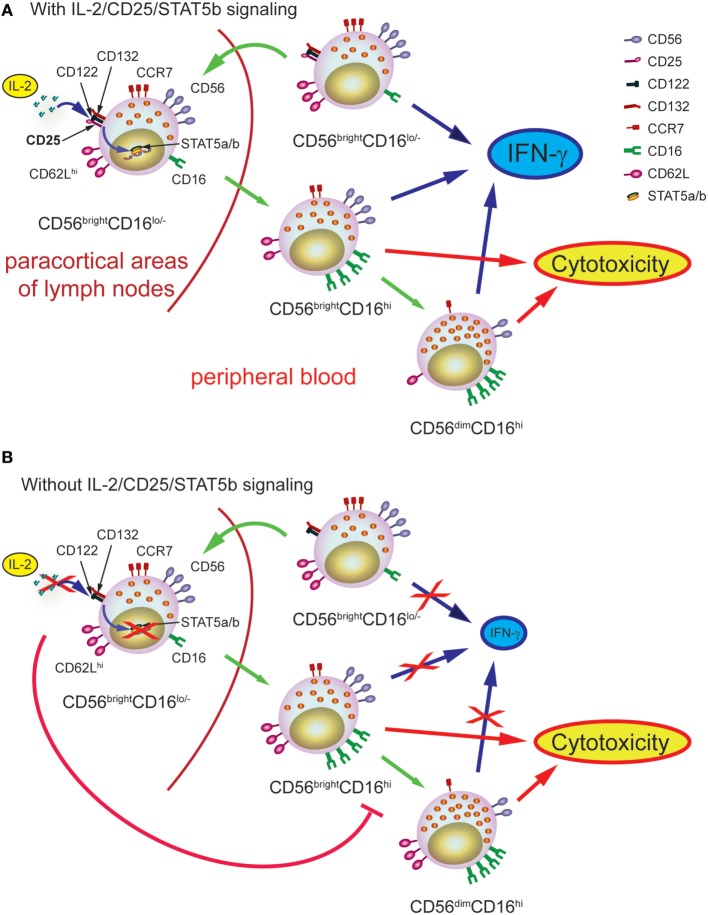
Schematic representation of the role of IL-2 during human natural killer (NK) cell maturation. **(A)** In healthy individuals, IL-2 promotes the maturation of CD56^bright^CD16^lo^ NK cells into CD56^bright^CD16^hi^ NK cells and then, into CD56^dim^CD16^hi^ NK cells. The first process occurs probably in the paracortical areas of the lymph nodes while the second process occurs once CD56^bright^CD16^hi^ NK cells left the lymph nodes and circulate in periphery, thanks to the imprint provided by IL-2. The transition from CD56^bright^CD16^lo^ NK cells into CD56^bright^CD16^hi^ NK cells and then, into CD56^dim^CD16^hi^ NK cells is accompanied by the downregulation of CCR7 and CD62L, the calibration of the normal content of pfp and granzyme B, and the acquisition of optimal effector functions (IFN-γ production and cytotoxic response). **(B)** In the absence of IL-2 signaling through the high affinity receptor, the transition from CD56^bright^CD16^lo^ NK cells into CD56^bright^CD16^hi^ NK cells and then, into CD56^dim^CD16^hi^ NK cells cannot occur normally and, consequently, NK cells cannot normally progress from CD56^bright^CD16^hi^ NK cells to CD56^dim^CD16^hi^ NK cells, resulting in the accumulation of CD56^bright^CD16^hi^ NK cells in peripheral blood. Moreover, in the absence of adequate IL-2 signaling, NK cells display increased content of pfp and granzyme B, proliferation, and expression of CD62L, and impaired IFN-γ production, suggesting that they achieve an incomplete maturation program.

In summary, taking advantage of two PID, we provide compelling evidence about the physiological role of the IL-2/CD25/STAT5b axis in human NK cell maturation from CD56^bright^CD16^lo/−^ to CD56^dim^CD16^hi^ NK cells. The lessons learned from the analysis of NK cells from these patients are summarized in Figure 7. Accordingly, IL-2-producing cells of the adaptive immune response constitute a differentiation checkpoint for NK cells.

## Ethics Statement

Studies have been approved by the institutional review committee from Hospital de Niños “Ricardo Gutiérrez” and informed consent of the parents of the participating subjects was obtained because they are minors.

## Author Contributions

MC, MB, and MG made substantial contributions to conception and design, acquisition of data, or analysis and interpretation of data, and reviewed the article critically. LB made substantial contributions to conception and design, analysis and interpretation of data, and reviewed the article critically. NZ made substantial contributions to conception and design, analysis and interpretation of data. MC and NZ wrote and corrected the article and gave final approval.

## Conflict of Interest Statement

The authors declare that the research was conducted in the absence of any commercial or financial relationships that could be construed as a potential conflict of interest.
